# Telerehabilitation for individuals with spinal cord injury in low-and middle-income countries: a systematic review of the literature

**DOI:** 10.1038/s41393-022-00797-8

**Published:** 2022-04-11

**Authors:** Rosie M. Solomon, Raju Dhakal, Stephen J. Halpin, Ram Hariharan, Rory J. O’Connor, Matthew Allsop, Manoj Sivan

**Affiliations:** 1 Academic Department of Rehabilitation Medicine, Faculty of Medicine and Health, University of Leeds, Leeds, UK; 2 Spinal Injury Rehabilitation Centre, Bhaisepati, Sanga, Kavre, Nepal; 3 National Demonstration Centre in Rehabilitation, Leeds Teaching Hospitals NHS Trust, Leeds, UK; 4 Princess Royal Spinal Injury Unit, Northern General Hospital, Sheffield Teaching Hospitals NHS Foundation Trust, Sheffield, UK; 5 Leeds Institute of Health Science, University of Leeds, Leeds, UK

**Keywords:** Patient education, Disability, Lifestyle modification, Preventive medicine, Quality of life

## Abstract

**Study design:**

Systematic review.

**Objective:**

To systematically review the evidence for the effectiveness of telerehabilitation as an intervention for people with spinal cord injury (SCI) in low-and middle-income countries (LMICs).

**Setting:**

Not applicable.

**Methods:**

MEDLINE (Ovid), Embase (Ovid), Pubmed and Global Health databases were used to identify studies published between 1946–2020 meeting the following criteria: (1) patients with SCI diagnosis; (2) in LMIC; (3) an outcome measuring clinical functional ability, quality of life or all-cause mortality reduction. The risk of bias in studies was graded using revised Cochrane risk-of-bias tool in randomised trials (RoB 2) and risk-of-bias tool in non-randomised trials (ROBINS-I). Evidence levels were graded with Grading of Recommendations, Assessment, Development and Evaluations (GRADE).

**Results:**

In total, 107 articles were identified from the initial search. After screening, five studies were included. Some significant improvements to quality of life and pressure ulcer management were observed, alongside some improvement in functional ability with suggested improvement to depression scores. Telerehabilitation alleviated participants’ sense of social isolation, improved satisfaction scores and assisted them to remember techniques for SCI management. Telerehabilitation was valued by health professionals. There was no reduction in all-cause mortality.

**Conclusion:**

There is insufficient evidence to recommend telerehabilitation as an intervention to treat and manage SCI in LMICs, although there is an indication of potential patient benefit. Further research is required to better understand the causal mechanisms underpinning the use of telerehabilitation and establish its efficacy, in the context of resource-limited settings.

## Introduction

The global incidence of SCI is estimated to be between 10.4 and 83 per million individuals per year [1], with a male predominance [1–7]. Such a large range demonstrates difficulty in collating epidemiological SCI data globally; particularly in LMICs as data collection is difficult due to lack of national trauma databases [1, 6, 8, 9]. SCI appears to be greater problem in LMICs; incidence is reportedly four times that in high-income countries [10] with a higher mortality rate [7]. The aetiology of SCI differs between countries; in LMIC, the primary cause is falls [6]; whereas Motor Vehicle Collisions (MVC) is the leading cause in high-income countries [11]. However, as motor usage has increased in LMICs, MVCs are becoming a common cause of SCI [6].

There are significant challenges for those in LMICs to survive in the community after discharge from hospital; patients face social isolation, poverty, depression and unemployment [3, 12–17]. Furthermore, limited access to and inadequate community care increases risk of complications. Those with SCI are more at risk of pressure ulcers and urinary tract infections which leads to deterioration, rehospitalisation and death [13, 14]. Most complications can be managed with simple, inexpensive treatments at home, such as education regarding positioning techniques to reduce and alleviate pressure provision of walking aids or appropriate antibiotics, according to numerous international clinical guidelines [18–20]. In studies performed in Nepal and Bangladesh, 25% and 20% of patients with SCI died within 2 years post-discharge from hospital, respectively [13, 14], most commonly from sepsis. There is an urgent need for better post-discharge long-term and care for those with SCI in LMICs [21].

An approach increasingly being used to extend access to care in LMICs, particularly when care delivery is challenged and in remote geographical locations [22, 23], is telemedicine. Telemedicine involves using information and communication technologies to provide care and education [24]. Telerehabilitation is a subset of telemedicine defined as the provision of rehabilitation services at a distance using telecommunication technology [25], incorporating prevention and treatment. Telerehabilitation works as an effective intervention in many fields in high-income countries [26–29], with a systematic review reporting significant improvements in patient outcomes in over 70% (*n* = 64) of included studies [29]. Similar improvements in patient outcomes from telerehabilitation use are emerging from LMICs [30–32]. Telerehabilitation may be an approach to address the current unmet needs of people with SCI reintegrating into the community, post-discharge from hospital in LMICs [3, 12–17]. A previous systematic review [33] has reported findings of randomised control trials (RCTs) of telerehabilitation for SCI across all settings. But no comprehensive search has been undertaken to date of primary research exploring the factors influencing uptake and impact of telerehabilitation. This systematic review will focus on the impact of telerehabilitation in LMICs on functional outcomes and quality of life for those with SCI, and factors related to its implementation.

## Methods

### Literature search strategy

The study protocol was registered with PROSPERO (registration number CRD42021232462). We identified relevant studies by conducting an electronic search of current literature using the databases MEDLINE and EMBASE via Ovid, and PUBMED and Global Health; 1946 to 2020. A comprehensive search strategy was developed, including MeSH and keywords for “telerehabilitation” AND “low-and middle-income country” AND “spinal cord injury” (Supplementary Table 1).

### Inclusion and exclusion criteria

#### Population

Patients were included if they had a SCI diagnosis and received telerehabilitation, in a clinical study, as an intervention for management or treatment of their condition. The clinical study occurred in a country defined as LMIC, at the time of intervention. LMICs were defined as a country belonging to the World Health Organisation (WHO) classification of a low or low-middle-income country at the time of the study [34].

#### Intervention

We included studies which involved rehabilitation using telecommunication methods. All telemedicine modalities were included: store-and-forward, remote monitoring and interactive services. All forms of telecommunication methods were included (i.e., telephone, Internet, video and audio conferencing).

#### Comparator

Comparator groups had SCI diagnosis and were enroled in the clinical studies in LMICs. They did not receive telerehabilitation as an intervention for the management or treatment of their condition. These groups received routine care or received some minor additional educational tools, to assist management of their SCI.

#### Outcomes

We included studies which reported the effect of telerehabilitation on any long-term clinical quantitative or qualitative outcome. Outcomes included functional independence scores, quality of life and all-cause mortality. Secondary outcomes such as medical complications, economic analysis or perspectives of telerehabilitation were included.

#### Study design

Primary research studies were included. These included RCTs, pilot study, prospective study, retrospective study and case series. Reviews, single case studies, editorial reports and protocols were excluded.

### Selection process

Articles were identified using the search strategy. Following removal of duplicated results, the identified articles were screened using the title and abstract. Articles were included or excluded in line with the outlined criteria. The full text was used to rescreen the articles. We assessed the methodological quality of articles before inclusion in the systematic review. Included articles were then critically appraised. Two researchers performed every stage independently (RMS and MS). In cases of discrepancy, a third researcher was consulted (MJA).

### Quality assessment

We assessed the risk of bias of studies included in this review using the RoB 2 [35] and ROBINS-I tools [36], for randomised and non-randomised studies respectively. We then assessed the overall certainty in the evidence with GRADE criteria [37, 38]. Two researchers independently assessed each study, using the tools (RMS and MS). In cases of discrepancy, a third researcher was consulted for resolution (MJA).

### Data collection and analysis

Data from studies was extracted and stored in a Microsoft Excel Spreadsheet. The data were then summarised and presented in tables. A narrative framework [39] was used for the qualitative part of the mixed-methods study. To represent the state of existing literature and underlying evidence, we developed a logic model which was created through intervention mapping [40]. The pathways across different components were mapped, reflecting inputs, interventions, participants and outcomes which were gathered and displayed. Outputs were demonstrated with different fonts to demonstrate evidence levels. Reporting is aligned with the Preferred Reporting Items for Systematic Reviews and Meta-analysis (PRISMA) statement [41]. The PRISMA checklist is shown in Supplementary Information.

## Results

**Fig. 1:**
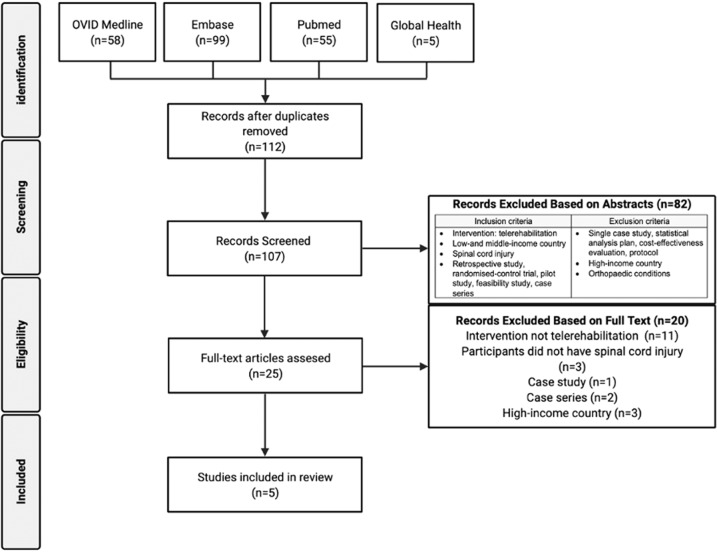
PRISMA flow chart for the literature. The number of articles identified for inclusion at each stage are presented. Article excluded are presented with reasons for exclusion.

We identified 107 articles from the search, summarised in Fig. 1. After exclusion, five studies were deemed suitable for inclusion [42–46]. Tables 1 and 2 display overviews.

**Table 1:** Summary of procedures and results from studies using telerehabilitation as an intervention for SCI in LMICs.

Study Study Design	Country Economic status Number of participants Length of study	RoB 2 [34]	ROBINS-I [35]	GRADE Quality [36, 37]	Procedure					Results	
					Intervention Procedure Sample size Age (years) Sex *n* (%) Diagnosis	Control Procedure Sample size Age (years) Sex *n* (%) Diagnosis	Professional delivering care	Method	Outcome Measures	Improved outcome	Statistical significance
Arora et al. [41] RCT	India and Bangladesh Low-Middle *n* = 120 12 weeks	Low	–	Moderate	12x weekly telephone advice: management of PU Informative pamphlet *n* = 60 Mean (*σ*) 35 (12) Male 52 (87) Female 8 (13)	Informative pamphlet only *n* = 60 Mean (*σ*) 36 (12) Male 54 (90) Female 6 (10)	Trained nurse or physiotherapist	Telephone consultation	Primary: size of PU at 12 weeks (cm^2^) Secondary: PUSH, Depth of PU, Undermining distance of PU, Braden score, HADS, Participation items—WHODAS, Utility score—EQ-5D-5L, Self-rated health EQ-5D-VAS, Participants’ impression of PU status, Participants’ confidence to manage PU, Clinician’s impression of PU status, Participants’ satisfaction, Self-report time for PU resolution	Mean adjusted between-group difference: Primary: 2.3 cm^2^ favouring intervention group Secondary: PUSH, Braden Score, WHODAS, Utility score—EQ-5D-5L, Self-rated health EQ-5D-VAS, Participants’ impression of PU status, Participants’ confidence to manage PU, Participants’ satisfaction All favoured intervention group	Primary: (95% CI −0.3–4.9; *p* = 0.08) Secondary: 8/13 statistically significant difference: PUSH, Braden Score, WHODAS, Utility score—EQ-5D-5L, Self-rated health EQ-5D-VAS, Participants’ impression of PU status, Participants’ confidence to manage PU, Participants’ satisfaction
Hossain et al. [42] RCT	Bangladesh Low-Middle *n* = 410 2 years	Low	–	Moderate	Pictorial educational booklet 1st year 26x two-weekly telephone advice 2nd year 12x monthly telephone advice Provided with $AU80 for miscellaneous items as required *n* = 204 Median (IQR) 33.4 (25.7 to 45.0) Male 181 (89%) Female 23 (11%)	Pictorial educational booklet only *n* = 206 Median (IQR) 31.4 (24.5 to 41.0) Male 188 (91%) Female 18 (9%)	Physiotherapist	Telephone consultation and home visit	Primary: all-cause mortality rate Secondary: SCI-SCS, PUSH, CESD-R, WHODAS, SF12 PCS, SF12 MCS, SCIM-SR, PU status, Bed-bound, House-bound, Unemployed	Primary: Intervention group 15/204 (7.4%) died. Control group 16/206 (7.8%) died Secondary: No clear between group differences	Primary: hazard ratio from unadjusted Cox model = 0.93 (95% CI, 0.46–1.89; *p* from log rank test 0.85) Secondary: No statistically significant difference between outcomes in intervention
Hossain et al. [43] Pilot RCT	India Low *n* = 30 2 years	Unclear	–	Low	1st year 26x two-weekly telephone advice 2x home visit 2nd year 12x monthly telephone advice 1x home visit *n* = 15 Median (IQR) 29 (24–35) Male 13 (87) Female 2 (13)	Standard care 1 telephone call 1 home visit, at-risk patients If participant rang centre, received advice *n* = 15 Median (IQR) 34 (23–36) Male 13 (87) Female 2 (13)	Physiotherapist	Telephone consultation and home visit	Primary: all-cause mortality rate Secondary: SCI secondary conditions scale, Presence of PU, PUSH, CES depression scale, SF12 PCS, SF12 MCS, SCIM, WHODAS, number of days out of bed in past week, number of days out the house in the past week, number of days working in the past week	Primary: 2 participants died (one in each group) Secondary: No clear between group differences	No statistical analysis performed
Tyagi et al. [44] CS	India Low *n* = 2 2 weeks	–	Serious risk of bias	Very low	Initial 4 weeks: no intervention. SCIM assessed Proceeding 4 weeks: 5x per week video-clips and guidance *n* = 2 Mean (*σ*) 40 (4.5) Male 1 (50%) Female 1 (50%) Participants: - SCI	–	Rehabilitation doctor and care team	Video-clips and guidance	Primary: SCIM	Primary: Initial 4 weeks: SCIM declined Participant 1: 14/40 Participant 2: 5/40 Proceeding 4 weeks (implementation of intervention): SCIM increased Participant 1: 27/40 Participant 2: 16/40	No statistical analysis performed
Leochico et al. [45] CS	Philippines Low-Middle *n* = 2 1 session	–	Serious risk of bias	Very low	Wheelchair follow-up via smartphone using Viber^TM^ *n* = 2 Mean (*σ*) 28 (0) Male 1 (50%) Female 1 (50%)	–	Rehabilitation doctor	Video call	Primary: Perspective of telerehabilitation Quantitative: Numerical questionnaire pre- and post-telerehabilitation intervention Qualitative: Interview	Primary: Quantitative: No improvement to score Qualitative: Perceptions of telerehabilitation improved	No statistical analysis performed

No participant feedback was available in studies which did not perform qualitative interviews or analysis.

*RCT* randomised control trial, *MM* mixed-methods study, *CS* case series, *σ* standard deviation, *IQR* interquartile range, *SCI* spinal cord injury, *PU* pressure ulcer, *ICT* information and communication technology, *CRP* Centre for the Rehabilitation of the Paralysed, *FEV1* Forced Expiratory Volume in 1 s, *PEF* peak expiratory flow, *WHOQOL-BREF* brief version of WHO quality of life scale, *ESES* Exercise Self-Efficacy Scale, *PUSH* Pressure Ulcer Scale for Healing (measures severity of pressure ulcer), *HADS* Hospital Anxiety and Depression Score, *WHODAS* World Health Organisation Disability Assessment Schedule (participation over the past 30 days), *EQ-5D-5L* Euro Quality of Life 5-dimensional 5-level (measures health state), *VAS* Visual Analogue Scale, *SCI-SCS* Spinal Cord Injury Secondary Conditions Scale, *CESD-R* Centre for Epidemiological Studies Depression Scale revised version, *PCS* Physical Component Score, *SF12* Short Form Health Survey-12 (measures health related quality of life), *MCS* Mental Component Score, *SCIM* Spinal Cord Independence Measure, *MDT* multi-disciplinary team, *CES* Centre for Epidemiological studies.

**Table 2:** Summary of results from studies using telerehabilitation as an intervention for SCI in LMICs.

Study	RoB 2 [34]	ROBINS-I [35]	GRADE Quality [36, 37]		Results	
				Outcome measures	Outcome	Statistical significance
Arora et al. [41]	Low	–	Moderate		Mean adjusted between-group difference:	95% CI; *p* value:
				Primary:	Primary:	Primary:
				size of PU at 12 weeks (cm^2^)	2.3	−0.3–4.9; *p* = 0.08
				Secondary:	Secondary:	Secondary:
				PUSH score /17	1.8	0.3–3.3; *p* = 0.02
				Depth of PU (cm)	0.2	−0.1–−0.5; *p* = 0.17
				Undermining distance of PU (cm)	0.6	−0.2–1.4; *p* = 0.14
				Braden score (23 points	1.4	0.7–2.0; *p* < 0.001
				Depression items—HADS (21 points)	0.2	−1.1–1.4; *p* = 0.77
				Participation items—WHODAS (40 points)	2.3	0.8–3.8; *p* = 0.003
				Utility score—EQ-5D-5L (units)	0.1	0.02–0.2; *p* = 0.01
				Self-rated health EQ-5D-VAS (100 points)	10.5	4.5–16.6; *p* = 0.001
				Participants' impression of PU status (10 points)	0.8	−0.1–1.7; *p* = 0.08
				Participants' confidence to manage PU (10 points)	1.7	1.0–2.3; *p* < 0.001
				Clinician’s impression of PU status (10 points)	0.6	−0.3–1.4; *p* = 0.18
				Participants' satisfaction (10 points)	2.1	1.3–2.8; *p* < 0.001
				Self-report time for PU resolution	Self-report time for PU resolution	Self-report time for PU resolution
Hossain et al. [42]	Low	–	Moderate	Primary:	Primary:	Primary: Hazard ratio from unadjusted Cox model
				All-cause mortality rate	Intervention group 15/204 (7.4%) died Control group 16/206 (7.8%) died	0.93 (95% CI, 0.46–1.89; *p* from log rank test 0.85)
				Secondary Continuous Outcomes:	Secondary: Continuous Outcomes (Adjusted between-group differences):	Secondary: Continuous Outcomes (95% CI; *p* value):
				SCI-SCS (/40)	−0.3	−0.8–0.3; *p* = 0.39
				PUSH (/17)	−0.2	−0.9–0.6; *p* = 0.69
				CESD-R (/60)	0.0	−2.1–2.1; *p* = 1.00
				WHODAS (/40)	0.2	−0.8–1.2; *p* = 0.69
				SF12 PCS	0.7	−0.3–1.8; *p* = 0.18
				SF12 MCS	−0.1	−2.6–2.4; *p* = 0.94
				SCIM-SR (/100)	1.3	−1.0–3.6; *p* = 0.27
				Binary Outcomes:	Binary Outcomes (Effect calculated with log-binomial regression):	Binary Outcomes: (Adjusted risk ratio)
				PU status	0.92	0.56–1.53
				Bed-bound	0.80	0.22–2.91
				House-bound	0.81	0.52–1.14
				Unemployed	1.02	0.92–1.13
Hossain et al. [43]	Unclear	–	Low	Primary: All-cause mortality rate Secondary: SCI secondary conditions scale (/49) Presence of PU (*n*) PUSH (/17) CES depression scale (/60) SF12 PCS SF12 MCS SCIM (/100) WHODAS (/40) Number of days out of bed in past week Number of days out the house in the past week Number of days working in the past week	Primary: Intervention group 1/15 (6.7%) died Control group 1/15 (6.7%) died Secondary: No clear between group differences	No statistical analysis performed
Tyagi et al. [44]	–		Very low	SCIM (/40)	Initial 4 weeks: Participant 1: 14/40 Participant 2: 5/40 Proceeding 4 weeks (implementation of intervention): SCIM increased Participant 1: 27/40 Participant 2: 16/40	No statistical analysis performed
Leochico et al. [45]	–		Very low	Primary: Perspective of telerehabilitation Quantitative: Numerical questionnaire pre- and post-telerehabilitation intervention Qualitative: Interview	Primary: Quantitative: No improvement to score Qualitative: Perceptions of telerehabilitation improved following telerehabilitation use	No statistical analysis performed

No participant feedback was available in studies which did not perform qualitative interviews or analysis.

*SCI* spinal cord injury, *PU* pressure ulcer, *ICT* information and communication technology, *CRP* Centre for the Rehabilitation of the Paralysed, *FEV1* Forced Expiratory Volume in 1 s, *PEF* peak expiratory flow, *WHOQOL-BREF* brief version of WHO quality of life scale, *ESES* Exercise Self-Efficacy Scale, *PUSH* Pressure Ulcer Scale for Healing (measures severity of pressure ulcer), *HADS* Hospital Anxiety and Depression Score, *WHODAS* World Health Organisation Disability Assessment Schedule (participation over the past 30 days), *EQ-5D-5L* Euro Quality of Life 5-dimensional 5-level (measures health state), *VAS* Visual Analogue Scale, *SCI-SCS* Spinal Cord Injury Secondary Conditions Scale, *CESD-R* Centre for Epidemiological Studies Depression Scale revised version, *PCS* Physical Component Score, *SF12* Short Form Health Survey-12 (measures health related quality of life), *MCS* Mental Component Score, *SCIM* Spinal Cord Independence Measure, *MDT* multi-disciplinary team, *CES* Centre for Epidemiological studies.

### Location and study design

The studies took place in India [42, 44, 45], Bangladesh [43] and Philippines [46]. The median (interquartile range) sample size was 30 participants (263). Two studies [42, 43] were RCTs, there was one pilot RCT [44] and two studies were case series [45, 46].

### Analysis

The studies were heterogenous in terms of their evidence levels, study population size, study design, intervention and outcome measures, preventing the ability to undertake a meta-analysis.

### Effectiveness of telerehabilitation for SCI

Three studies measured functional independence using spinal cord independence measure (SCIM) [43–45]. SCIM improved for participants who received telerehabilitation in the case series [45] (*n* = 2). However, in the RCT and the pilot RCT which measured SCIM, no improvements were found [43, 44].

Three studies investigated quality of life [42–44]. In an RCT [42], Euro Quality of Life Visual Analogue Scale and EQ-5D-5L [47, 48] both improved with statistical significance. The Short form 12- (SF12), did not demonstrate improvements to quality of life in the intervention group of an RCT [43] and pilot RCT [44].

Telerehabilitation was not found to significantly reduce all-cause mortality rate in an RCT (*n* = 410) [43] or pilot RCT (*n* = 30) [44].

In an RCT, patients receiving telerehabilitation had less severe pressure ulcers and a decreased chance of a new sore developing [42], than control participants. Additionally, those receiving telerehabilitation were found to be more satisfied [42].

Depression was investigated in three studies [42–44]. Some minor improvement was seen to depression score, but without statistical significance [42].

### Experiences of telerehabilitation implementation

A summary of the qualitative experiences of telerehabilitation from participants and health professionals providing care is summarised in Supplementary Table 2. Participants and health professionals reported positive experiences with telerehabilitation. For participants, such benefits included avoiding stress associated with waking up early or waiting in line outside the clinic, not having to travel and become fatigued, reduced expenses and having immediate and direct communication with an experienced doctor [46]. Telerehabilitation alleviated a sense of social isolation and feelings of depression for some participants. Talking to health professionals made participants “feel good”. Patient participants built rapport with their provider, who they reported having trust and confidence in [49]. Health professionals had increased confidence in care provision and worked with participants to set goals [49].

Difficulties in the implementation of telerehabilitation interventions were reported. These included technical issues using the technology and issues with internet connectivity [45]. Studies adjusted their methodology to support patients; for example, using telephone calls instead of video [46]_._ The health professionals providing care expressed some concern. In Bangladesh, professionals felt “hopeless if pressure ulcers became severe” [49]. They also described how participants occasionally under-reported pressure injuries and it was difficult to assess the seriousness of the pressure ulcer [49]. The professionals explained that many participants did not have access to a smartphone to take photographs or could not afford to send images using their mobile phone.

Explanations for the lack of improvement to functional ability and lack of reduction in all-cause mortality rate using telerehabilitation were proposed by a process evaluation [49] in relation to one of the included studies [43]. It concluded that telerehabilitation alone was unable to solve the economic and social problems faced by people with SCI in Bangladesh. Some participants were unable to adhere to the advice given during the telerehabilitation consultation. For example, participants with pressure ulcers were unable to remain on bedrest, as suggested, because they needed to work and support their families [49]; similarly reported in an included RCT [42]. Some participants could not adhere to advice because they were home alone during the day and lacked family/friend support to care for their pressure ulcers or change bed linen to remain dry [42]. Other participants could not follow advice because they did not have access to or could not afford resources, including basic dressings or adequate nutrition [42]. Many faced significant financial difficulties, even those in the study which provided a small sum of money (~£40) to assist with purchasing basic equipment (e.g., bladder supplies) [49]. Participants were unable to address their monetary difficulties through work. In one study, participants were provided with some vocational training to help them to find jobs when they returned to the community [43]. Yet, there were few work opportunities for them [49].

### Quality of evidence

Risk of bias scores were variable. The main sources of bias were selection bias, attrition bias and bias due to poor classification of the procedures for intervention and control participants. Two studies had low RoB 2 scores and moderate GRADE scores [42, 43]. One study had an unclear RoB 2 score and a low GRADE score [44]. Two studies had unclear risk of bias ROBINS-I scores and very low GRADE scores [45, 46].

## Discussion

This systematic review evaluated existing literature about the effectiveness of telerehabilitation as an intervention for the management of SCI, focusing on LMICs. Five studies were identified, with no interventions or outcomes similar enough for pooling of data. Of the five included studies, there is some suggestion that telerehabilitation improves the lives of those with SCI in LMICs. Some significant improvements to quality of life and pressure ulcer management were observed, alongside some improvement in functional ability and suggested improvement to depression scores. Accompanying qualitative data suggests telerehabilitation alleviated participants’ sense of social isolation, alleviated feelings of depression, improved satisfaction scores and assisted them to remember techniques for SCI management. Telerehabilitation was valued by health professionals and participants. There was no reduction in all-cause mortality. Notably, none of the studies reported that participants had difficulty using the equipment, aligning with telemedicine usage broadly [23]. There was very limited information on the technical feasibility of implementation and use of telerehabilitation from the perspective of professionals, which could be explored in future studies. Telerehabilitation should support health care systems to provide the best care for patients. From embedded process evaluations and qualitative interviews, the key factors influencing engagement with telerehabilitation were its ability to allow direct contact with an experienced professional for guidance and support, especially regarding pressure ulcer management, and improving daily functioning. Additionally, telerehabilitation assisted rapport building between participants and health professionals.

The WHO stated digital interventions should not substitute care, but should strengthen health systems [22]. Efforts to improve policy and develop rehabilitation care are necessary in LMICs. Telerehabilitation will only ever be able to enhance existing provision of good-quality care, but not used in place of it. The process evaluation [49], which was performed in relation to an included study [43], reported that during telerehabilitation consultations, participants were screened for key complications and referred to local service providers, where necessary. Yet such services were often unavailable or inaccessible. Alternatively, health professionals would try to refer participants back to the specialist rehabilitation centre from which they received their initial care. However, often there was not an available bed for the participant; or if there was, the participant would not be able to afford to travel. Here, telerehabilitation is being used alone and, for some participants, is failing to resolve unmet rehabilitation needs due to the weaker health systems in which they were being used. Vital local services and rehabilitation care must be adequate and available in order for those with SCI to benefit from telerehabilitation.

The studies were heterogenous in terms of their design and outcomes used, making comparison difficult. There is a need to establish a set of outcomes for the investigation of telerehabilitation. Supplementary Fig. 1 demonstrates a logic model to conceptualise and describe the current understanding of telerehabilitation as an intervention and demonstrates possible changes that may arise from its use, based on the included evidence. Many studies did not demonstrate significant differences to clinical outcomes between intervention and control participants. Yet, the researchers wrote they believed telerehabilitation to have holistically positive effects. For example, telerehabilitation alleviated participants’ sense of social isolation and feelings of depression when returning to the community, making them “feel good” [49]. Additionally, telerehabilitation increased health professional’s confidence in care delivery [49]. Given rehabilitation involves improving functional ability and quality of life [50], it is important that the outcome measures truly reflect this. The need to better define causal mechanisms underpinning any positive and negative findings about telerehabilitation use in LMICs is evident from the logic model (Supplementary Fig. 1). Mapping and understanding the causal mechanisms of an intervention are vital to develop intervention use [51]. Accompanying process evaluations with subsequent telerehabilitation development may help to derive understanding about whether, how and why the intervention is achieving its intended effect. This will help to refine the use of telerehabilitation and aid its implementation in the context of LMICs. Furthermore, in 2011, WHO released a consensus statement highlighting the need for rigorous evaluation to generate evidence about eHealth [52]. There is a need for good-quality studies which follow CONSORT guidelines, in order to ensure confidence in the findings. Largely absent from the literature are considerations of cost relating to telerehabilitation in LMICs. There is a lack of economic analysis of eHealth generally [26, 53–55]. The included studies reflect this, with only one RCT [42] reporting a cost-effectiveness analysis [56]. There is a need to further define economic models reflecting the technology and context of telerehabilitation approaches in low resource settings.

### Limitations

Our review included a comprehensive search strategy which was used to search several databases. However, we acknowledge that due to time and resource constraints we were unable to search grey literature sources and consequently findings may not reflect the entirety of research literature on telerehabilitation in LMICs.

## Conclusion

Limited literature is available reporting the use and effectiveness of telerehabilitation as an intervention for SCI in LMICs. Whilst feasible, telerehabilitation did not consistently significantly improve outcomes in patients with SCI, including functional ability and all-cause mortality reduction. While impact varied for patients, telerehabilitation is acceptable to health professionals, care-providers and participants. There is a need for better modelling of the causal mechanisms underpinning telerehabilitation. Additionally, there is a need to establish the intended outcomes to be investigated for telerehabilitation, to guide approaches to evaluation of SCI in LMICs.

## Supplementary information

Supplementary table 1 Search strategy

Supplementary Table 2 Qualitative findings arranged into themes

Supplementary Fig. 1 Logic Model to conceptualise Telerehabilitation to manage and improve outcomes in those with spinal cord injury in low-and middle-income countries

## Data Availability

All data extracted from the included studies are available in original publications.
