# Supplementary material for: Telerehabilitation for individuals with spinal cord injury in low-and middle-income countries: a systematic review of the literature
Source: Spinal Cord. 2022 Apr 11;60(5):395–403. doi: 10.1038/s41393-022-00797-8 (PMC9106582; doi:10.1038/s41393-022-00797-8)
Supplement: Supplementary file 1 — Supplementary table 1 Search strategy [file 41393_2022_797_MOESM1_ESM.docx]

**Supplementary Table 1** The comprehensive search strategy used, in this systematic review, to identify relevant studies

| Search term | Search |
| --- | --- |
| “Low-and middle-income country” | **((afghanistan or albania or algeria or american samoa or angola or "antigua and barbuda" or antigua or barbuda or argentina or armenia or armenian or aruba or azerbaijan or bahrain or bangladesh or barbados or republic of belarus or belarus or byelarus or belorussia or byelorussian or belize or british honduras or benin or dahomey or bhutan or bolivia or "bosnia and herzegovina" or bosnia or herzegovina or botswana or bechuanaland or brazil or brasil or bulgaria or burkina faso or burkina fasso or upper volta or burundi or urundi or cabo verde or cape verde or cambodia or kampuchea or khmer republic or cameroon or cameron or cameroun or central african republic or ubangi shari or chad or chile or china or colombia or comoros or comoro islands or iles comores or mayotte or democratic republic of the congo or democratic republic congo or congo or zaire or costa rica or "cote d’ivoire" or "cote d’ ivoire" or cote divoire or cote d ivoire or ivory coast or croatia or cuba or cyprus or czech republic or czechoslovakia or djibouti or french somaliland or dominica or dominican republic or ecuador or egypt or united arab republic or el salvador or equatorial guinea or spanish guinea or eritrea or estonia or eswatini or swaziland or ethiopia or fiji or gabon or gabonese republic or gambia or "georgia (republic)" or georgian or ghana or gold coast or gibraltar or greece or grenada or guam or guatemala or guinea or guinea bissau or guyana or british guiana or haiti or hispaniola or honduras or hungary or india or indonesia or timor or iran or iraq or isle of man or jamaica or jordan or kazakhstan or kazakh or kenya or "democratic people’s republic of korea" or republic of korea or north korea or south korea or korea or kosovo or kyrgyzstan or kirghizia or kirgizstan or kyrgyz republic or kirghiz or laos or lao pdr or "lao people's democratic republic" or latvia or lebanon or lebanese republic or lesotho or basutoland or liberia or libya or libyan arab jamahiriya or lithuania or macau or macao or "macedonia (republic)" or macedonia or madagascar or malagasy republic or malawi or nyasaland or malaysia or malay federation or malaya federation or maldives or indian ocean islands or indian ocean or mali or malta or micronesia or federated states of micronesia or kiribati or marshall islands or nauru or northern mariana islands or palau or tuvalu or mauritania or mauritius or mexico or moldova or moldovian or mongolia or montenegro or morocco or ifni or mozambique or portuguese east africa or myanmar or burma or namibia or nepal or netherlands antilles or nicaragua or niger or nigeria or oman or muscat or pakistan or panama or papua new guinea or new guinea or paraguay or peru or philippines or philipines or phillipines or phillippines or poland or "polish people's republic" or portugal or portuguese republic or puerto rico or romania or russia or russian federation or ussr or soviet union or union of soviet socialist republics or rwanda or ruanda or samoa or pacific islands or polynesia or samoan islands or navigator island or navigator islands or "sao tome and principe" or saudi arabia or senegal or serbia or seychelles or sierra leone or slovakia or slovak republic or slovenia or melanesia or solomon island or solomon islands or norfolk island or norfolk islands or somalia or south africa or south sudan or sri lanka or ceylon or "saint kitts and nevis" or "st. kitts and nevis" or saint lucia or "st. lucia" or "saint vincent and the grenadines" or saint vincent or "st. vincent" or grenadines or sudan or suriname or surinam or dutch guiana or netherlands guiana or syria or syrian arab republic or tajikistan or tadjikistan or tadzhikistan or tadzhik or tanzania or tanganyika or thailand or siam or timor leste or east timor or togo or togolese republic or tonga or "trinidad and tobago" or trinidad or tobago or tunisia or turkey or "turkey (republic)" or turkmenistan or turkmen or uganda or ukraine or uruguay or uzbekistan or uzbek or vanuatu or new hebrides or venezuela or vietnam or viet nam or middle east or west bank or gaza or palestine or yemen or yugoslavia or zambia or zimbabwe or northern rhodesia or global south or africa south of the sahara or sub-saharan africa or subsaharan africa or africa, central or central africa or africa, northern or north africa or northern africa or magreb or maghrib or sahara or africa, southern or southern africa or africa, eastern or east africa or eastern africa or africa, western or west africa or western africa or west indies or indian ocean islands or caribbean or central america or latin america or "south and central america" or south america or asia, central or central asia or asia, northern or north asia or northern asia or asia, southeastern or southeastern asia or south eastern asia or southeast asia or south east asia or asia, western or western asia or europe, eastern or east europe or eastern Europe)** |
| “Telecommunication” | **((videophone? or video-phone? or (videoconferen* or video-conferen*) or ((transfer* or transmi*) adj3 (image? or radiogra* or photo* or picture? or x-ray?)) or tele* or medical record system* or hotline? or (etherap* or e-therap*) or ("e medicine*" or "emedicine*") or electronic mail* or (ehealth* or e-health*) or (remote* adj4 (health* or consult* or counsel* or monitor* or therap* or treatment?)) (distan adj4 (health* or consult* or counsel* or monitor* or therap* or treatment?)) ((mobile* or phone? or telephone?) adj3 (follow up* or support* or interview*)) ((mobile* or phone? or telephone?) adj3 (consult* or counsel*)) or answering service? or Telemetry/ or *Hotlines/ or Local Area Networks/ or *Internet/ or Computer Communication Networks/ or Videoconferencing/ or Microscopy, Video/ or Television/ or Modems/ or Cellular Phone/ or Answering Service/ or Telephone/ or Teleradiology/ or Telepathology/ or Remote Consultation/ or Telemedicine/ or Telefacsimile/ or Satellite Communications/ or Radio/ or Radar/ or Electronic Mail/ or Telecommunications/))** |
| “Spinal cord injury” | **(exp SPINAL CORD ISCHEMIA/ or exp CENTRAL CORD SYNDROME/ or (myelopathy adj3 (traumatic or post-traumatic)) or (spinal cord adj3 (contusion or laceration or trauma or injur* or ischemi*)) or ((spine or spinal) adj3 (broken or break* or fracture* or wound* or trauma* or injur* or damag*)) or (tetraplegia* or tetraplagi* or tetraparesis) or (quadriplegi* or quadriparesi*) or (paraplegi* or paraparesis) or exp QUADRIPLEGIA/ or exp PARAPLEGIA/ or exp SPINAL FRACTURES/ or exp SPINAL CORD INJURIES/)** |
