# Supplementary material for: Telerehabilitation for individuals with spinal cord injury in low-and middle-income countries: a systematic review of the literature
Source: Spinal Cord. 2022 Apr 11;60(5):395–403. doi: 10.1038/s41393-022-00797-8 (PMC9106582; doi:10.1038/s41393-022-00797-8)
Supplement: Supplementary file 2 — Supplementary Table 2 Qualitative findings arranged into themes [file 41393_2022_797_MOESM2_ESM.docx]

**Supplementary Table 2** Qualitative findings arranged into themes

| Level | Cost and resources | Technical Difficulties | User behaviour |
| --- | --- | --- | --- |
| Patient level | Did not have basic resources: access to basic dressings, adequate nutrition, appropriate bed overlays or pressure cushions (41)  Did not have enough money to buy resources (48).  Did not have smartphone required to take and send photos (48)**.**  No job opportunities so had difficulty making money (48)**.**  Unable to afford travel fare to return to specialist centre as required to manage complications (48)**.** | Problems with technology.  Slow internet speed: had to send photos rather than videos (44), other techniques such as phone call, text messaging and online asynchronous technique like chat messaging were used (45). | Increased satisfaction with care (41).  Talking to professional made them “feel good” (48).  Improved attitude towards telerehabilitation throughout study (45).  Perceived benefits included no fatigue from travel, no stress in waking up and early and waiting in line outside the clinic, reduced expenses, instant and direct communication with a doctor and less burden on family (45).  Alleviated social isolation and feelings of depression (48).  Intervention reminded users about physiotherapy exercises (45) and pressure ulcer management, other complication management (48).  Intervention clarified instructions: practical home exercises were demonstrated through synchronous video call (45).  Not enough support from family/friends: home alone during the day so did not have the support to regularly dress pressure ulcers or change bed linen to remain dry (41).  Had to work to provide for family (41, 48).  Under-reported severity of problems (48)**.** |
| Health service provider level | Care provision inadequate to support participants: local services unable to manage patients and specialist care centres did not have bedspace required (48).  Management of secondary conditions inadequate, perpetuating complications: conditions such as diabetes were not properly managed, so pressure ulcers were uncontrolled. Did not have access to surgery and wound debridement needed (41). |  | Confidence in managing participants increased (48)**.**  Able to offer advice about available opportunities (48)**.**  Professionals identified a patient’s wheelchair was inadequate through telerehabilitation and advised which model was required (45).  Built rapport with participants (48).  Screened participants for complications (48).  Worked with patients to help them set up a care plan, rather than dictating their actions and set goals:  aimed for better skin management and increased community and family participation (48). |
