# Supplementary material for: Telerehabilitation for individuals with spinal cord injury in low-and middle-income countries: a systematic review of the literature
Source: Spinal Cord. 2022 Apr 11;60(5):395–403. doi: 10.1038/s41393-022-00797-8 (PMC9106582; doi:10.1038/s41393-022-00797-8)
Supplement: Supplementary file 3 — Supplementary Fig. 1 Logic Model to conceptualise Telerehabilitation to manage and improve outcomes in those with spinal cord injury in low-and middle-income countries [file 41393_2022_797_MOESM3_ESM.docx]

**Supplementary Fig.1** Logic Model to conceptualise Telerehabilitation to manage and improve outcomes in those with SCI in LMICs, built from examining the included published literature.

The model illustrates the pathway between inputs required for telerehabilitation interventions and intended impact. It reads from left to right, with a typology of inputs in the first column, the interventions and participants in the next two columns, short-, medium- and long-term outcomes follow. The model indicates (via differing text types) where there was stronger or weaker evidence of links in the pathway.

**Outputs**

*Activities Participation*

**Outcomes**

*Short Medium Long*

**Inputs**

- Improve participant attitudes
- Improve health professional attitudes
- Technology

Reduce complications:

- PRESSURE ULCERS
- Depression
- Health professionals
- Phone consultations
- Those with SCI

Rev. 7/09

- IMPROVE QUALITY OF LIFE
- Enable participants to use technology
- Family or friend support

**Th e**

- Reduce social isolation
- Enable participants to engage in telerehabilitation
- Improve functional ability

**External Factors: Difficult to assess severity of illness, difficulty in using digital technology especially for those with disabilities, poor internet connectivity**

**Assumptions: Family/friend support available to assist patients, patients have a device to use**

- Asking participants to send in videos/photos

**Outcomes**

STRONG EVIDENCE = capital and underlined

STRONG EVIDENCE IN OPPOSING DIRECTION = capital

Weaker evidence = lower case and underlined

Weaker evidence that is conflicting = lower case

- Money
- SCI health service
